# Serum Interleukin (IL)-15 as a Biomarker of Alzheimer's Disease

**DOI:** 10.1371/journal.pone.0117282

**Published:** 2015-02-24

**Authors:** Ram J. Bishnoi, Raymond F. Palmer, Donald R. Royall

**Affiliations:** 1 Department of Psychiatry, University of Texas Health Science Center, San Antonio, Texas, United States of America; 2 Department of Family and Community Medicine, University of Texas Health Science Center, San Antonio, Texas, United States of America; 3 Department of Psychiatry, Family and Community Medicine, and Medicine, University of Texas Health Science Center, South Texas Veterans’ Health System Audie L. Murphy Division, Geriatric Research Education and Clinical Centers, San Antonio, Texas, United States of America; Nathan Kline Institute and New York Center for Dementia Research, United States

## Abstract

Interleukin (IL-15), a pro-inflammatory cytokine has been studied as a possible marker of Alzheimer’s disease (AD); however its exact role in neuro-inflammation or the pathogenesis AD is not well understood yet. A Multiple Indicators Multiple Causes (MIMIC) approach was used to examine the relationship between serum IL-15 levels and AD in a well characterized AD cohort, the Texas Alzheimer's Research and Care Consortium (TARCC). Instead of categorical diagnoses, we used two latent construct d (for dementia) and g’ (for cognitive impairments not contributing to functional impairments) in our analysis. The results showed that the serum IL-15 level has significant effects on cognition, exclusively mediated by latent construct d and g’. Contrasting directions of association lead us to speculate that IL-15’s effects in AD are mediated through functional networks as d scores have been previously found to be specifically related to default mode network (DMN). Our finding warrants the need for further research to determine the changes in structural and functional networks corresponding to serum based biomarkers levels.

## Introduction

Neuro-inflammation, mediated by both pro- and anti-inflammatory cytokines, in has been extensively reported in the Alzheimer’s disease (AD) literature [1,2]. Inflammatory processes have been linked to not only the pathogenesis of Alzheimer’s disease [3,4] but also with its associated emotional and behavioral symptoms [5,6]. Amyloid plaques are believed to accumulate acute phase proteins and cytokines, which are integral components of inflammatory processes that augment the harmful effects of Aβ [7]. There is overwhelming evidence that systemic immune response crosstalks with brain pathology. In response to injury, brain can mount a well regulated local immune response [8,9] and activate the peripherally lying immune cells [10,11]. Circulating cytokines are also known to cross the blood brain barrier (BBB) by saturable transport mechanism[12,13]. In a recent metaanalysis, levels of Interleukin (IL)-1β, IL-6, IL-12, tumor necrosis factor-α (TNF- α), and transforming growth factor-β (TGF-β) were reported to be elevated in the peripheral blood of individuals with AD compared with controls [2].

IL-15 is a pleiotropic and pro-inflammatory cytokine, is produced by activated blood monocytes, macrophages, dendritic cells, and activated glial cells [14,15]. In the Texas Alzheimer’s Research and Care Consortium (TARCC) cohort, serum levels of IL-15 were significantly and negatively related to total neuropsychiatric symptoms and symptom of hyperactivity in patients with AD [16]. In a cohort of AD patients, IL-15 was significantly related to basic activities of daily living (BADL) in AD patients in a gender dependent manner. Lower levels of IL-15 were related to greater functional dependence for males whereas increased levels of IL-15 were related to greater dependence for females [17].

IL-15 binds to its unique receptor, IL-15Rα, as well as two co-receptors Interleukin (IL)-2Rß and IL-2Rγ common chain. In addition to promoting T cell proliferation and inducing cytolytic effector cells, including natural killer and cytotoxic cells, IL-15 also stimulates B-cells to proliferate and secrete immunoglobulins [18–20]. IL-15 and its receptor (IL-15R) have been described in murine brain and cerebellum [21], as well as in fetal human brain [22]. Low levels of IL-15 were expressed by unstimulated human fetal astrocytes and microglia, and treatment of astrocytes with IL-1β, TNF-α, and Interferon (IFN)-γ increased the expression of IL-15 at both messenger RNA (mRNA) level and protein level [14]. The use of IL-15 activity neutralizing strategies is an efficient anti-inflammatory approach [23,24].

Unfortunately, little information is available on the exact role of IL-15 in neuro-inflammation and neuro-degeneration associated with AD. There are limited studies, with conflicting results where IL-15 was assessed as a marker of AD. In a small study, Rentzos et al found that AD patients had significantly higher cerebrospinal fluid IL-15 levels compared with patients with non-inflammatory neurological diseases [25]. In order to assess the role of IL-15 as a potential peripheral marker of immune reaction, Rentzos et al measured serum IL-15 levels in patients with AD, vascular dementia and healthy subjects. Contradicting the inflammatory hypothesis, they found lower serum levels in AD compared to healthy subjects and patients with vascular dementia and, treatment with acetylcholinesterase inhibitor (AChEI) had no influence on IL-15 concentrations in AD patients. These finding could not establish the role of IL-15 in AD pathogenesis [26].

Because of the difficulty, cost and invasiveness to obtain data from cerebrospinal fluid (CSF), recent research is focused on finding out the serum based biomarker for AD. In the present analysis, also using the TARCC dataset, we set out to explore the possibility that serum levels of IL-15 can assist in diagnosis of dementia. Instead of categorical diagnosis of dementia, we used a previously validated latent dementia phenotype (d), which represents the cognitive correlate of functional status. d is highly specific measure of dementia severity as measured by Clinical Dementia Rating Sum of Boxes (CDR-SB) [27] and accurately distinguishes AD and MCI with each other and controls [28,29]. We employed the Multiple Indicators and Multiple Causes (MIMIC) approach for our analysis, as recently applied to study the utility of serum Vitamin D Binding Protein (VDBP) as serum biomarker for AD [30].

## Materials and Methods

### 2.1 Subject cohorts

Subjects included N = 2016 TARCC participants [920 cases of Alzheimer’s disease (AD), 277 “Mild Cognitive Impairment “(MCI) cases, and 819 controls]. Each participant underwent a standardized annual examination that included a medical evaluation, neuropsychological testing, and clinical interview. Diagnosis of AD status was based on National Institute for Neurological Communicative Disorders and Stroke-Alzheimer’s Disease and Related Disorders Association (NINCDS-ADRDA) criteria [31]. Institutional Review Board approval was obtained at each site and written informed consent was obtained for all participants. The Institution Review Board (IRB) at Texas Tech University Health Sciences Center, Baylor College of Medicine, University of North Texas Health Science Center, the University of Texas Southwestern Medical Center, and the University of Texas Health Science Center—San Antonio approved this research.

### 2.2 Clinical Variables

The TARCC’s cognitive performance battery included digit span (WAIS-R, WAIS-III, WMS-R), Trail Making Test, WMS Logical Memory (WMS-R and WMS-III), Boston Naming Test (30- and 60-item versions), verbal fluency (FAS), Clock Drawing Test, the American National Adult Reading Test (AMNART), the Geriatric Depression Scale (GDS-30), mini-mental state examination (MMSE) [32], and the Clinical Dementia Rating scale (CDR) [27].

### 2.3 Assays

Non-fasting samples were collected in serum-separating tubes during clinical evaluations, allowed to clot at room temperature for thirty minutes, centrifuged, aliquoted, and stored at −80°C in plastic vials. Serum samples were sent frozen to Rules-Based Medicine (http://www.rulesbasedmedicine.com/, Austin, TX, USA) where they were thawed for assay without additional freeze-thaw cycles. Rules-Based Medicine conducted multiplexed immunoassay via their human multianalyte profile (human MAP). Assays conducted by this company utilizing this platform, including TARCC data, have been published elsewhere [33]. The TARCC’s serum biomarkers exhibit significant batch effects. The covariate-adjusted batch variables were used to adjust the observed serum biomarker values. Additionally, all observed variables and serum biomarkers were adjusted for age, gender, education, ethnicity, and APOE ε4.

### 2.4 Statistical Analyses

All the confirmatory factor analysis (CFA) and MIMIC analyses were performed using Analysis of Moment Structures software (AMOS) [34]. The analyses in this study were conducted in two major steps. First, the d was constructed by a bifactor confirmatory factor analysis in a SEM framework. Because cognitive performance is a weak predictor of functional outcomes, structural equation models were employed to explicitly distinguish functional status, and therefore "dementia-relevant" variance in cognitive task performance (i.e., d) from the variance that is unrelated to a dementing process (i.e., g'). d has been validated in multiple well characterized AD cohorts and can serve as dementia specific endophenotype [28,35,36].asAll observed measures, latent indicators and outcomes, were adjusted for age, gender, education, ethnicity and depression. Co-variances between the residuals were allowed to be estimated if they were significant and improved model fit. The latent variable of interest has been validated as predictors of observed TARCC outcomes in multivariate regression models and by receiver operating characteristic (ROC) analyses [28,37]. During this analysis, the missing biomarker and psychometric data were handled by Modern Missing Data Methods/Full information Maximum Likelihood (FIML) methods [38].

Second, MIMIC model was specified to investigate the potential association between IL-15 levels and dementia measures. Here, two latent variables (d & g’) intervene between a set of observed background variables (e.g. IL-15) predicting a set of observed response variables (cognitive and functional status measures) [39–41]. Three sets of relationships were evaluated: the measurement model, where the relationships between constituent items of the latent variable (cognitive and functional status measures) and the latent variables (d, g`) were observed; the structural regression equations, where the relationships among the latent variable and IL-15 were observed; and the direct effects, where the relationships between the constituent items of the latent variable and IL-15 were observed.

**Fit indices.** Model fit was assessed using four common test statistics: chi-square, the ratio of the chi-square to the degrees of freedom in the model (CMIN /DF), the comparative fit index (CFI), and the root mean square error of approximation (RMSEA). Where two nested models were compared, Akaike's Information Criterion (AIC) was added. A lower AIC statistic indicates better fit. A non-significant chi-square signifies that the data are consistent with the model [42]. However, in large samples, this metric is limited by its tendency to achieve statistical significance when all other fit indices (which are not sensitive to sample size) show that the model fits the data very well. A CMIN/DF ratio < 5.0 suggests an adequate fit to the data [43]. The CFI statistic compares the specified model with a null model [44]. CFI values range from 0 to 1.0. Values below 0.95 suggest model misspecification. Values approaching 1.0 indicate adequate to excellent fit. An RMSEA of 0.05 or less indicates a close fit to the data, with models below 0.05 considered “good” fit, and up to 0.08 as “acceptable” [45]. All fit statistics should be simultaneously considered when assessing the adequacy of the models to the data.

This analysis assess the utility of IL-15 as biomarker for AD by application of a linked MIMIC model, a special case of a longitudinal SEM, in which the influences of serum IL-15 levels on cognitive and functional measures of dementia are assessed simultaneously, through latent variables (d, g`) as well as the direct impacts. The direction of association between latent variables and reflective factors was hypothesized based on correlation observed in CFA model. Path coefficients (factor loadings) are the effect of the latent variable on its reflective indicators (apart from random errors, e), confirmed earlier in CFA model. Coefficients for the IL-15 levels are interpreted as are coefficients in regression analysis. Thus, for example, all other things equal, a statistically significant positive coefficient on a dichotomous indicator implies a higher mean value of d for the group identified by the 1L-15 levels. We hypothesized that 1L-15 levels in serum could predict the variability of d and also individual cognitive test performances.

There are several advantages to specifying and testing one multiple mediation model than several separate simple mediation models. First, testing the total indirect effect of IL-15 on cognitive measures is analogous to conducting a regression analysis with several predictors with the aim of determining the overall effect, if that exists. In our analysis, no significant direct effect was found. Second, it is possible to determine the extent to which mediators mediate the effect, conditional on the presence of other mediator in the model. Third, when multiple mediators are used in mediation models, the parameter bias can be removed.[46] Fourth, multiple mediator allows the determination of relative magnitude and direction of indirect effects associated with the mediators.

## Results

The demographic characteristics of the TARCC sample are presented in Table 1. The TARCC baseline sample is relatively highly educated, and has a slight preponderance of females. The AD group is significantly older, less well educated, and more impaired relative to controls on multiple measures.

**Table 1:** Descriptive Statistics (ANOVA).

			Post hoc tests			
	Variable N	Total Sample	AD	MCI	Controls	Main Effect
			N = 920	N = 277	N = 819	p
			Mean (SD)	Mean (SD)	Mean (SD)	
Gender (% female)	2016	59.6	56.6^♯^	54.5^♯^	64.7^†^ ^‡^	<0.001
Ethnicity (% Hispanic)	2015	26.6	16.0	41.8^†^	50.9^†^	<0.001
Age at Visit	2016	72.6 (9.4)	76.1 (8.3) ^‡^ ^♯^	73.4 (9.0)^†^ ^♯^	68.4 (9.1) ^†^ ^‡^	<0.001
Education	2016	13.9 (3.8)	14.2 (3.6)^♯^	13.5 (3.5)	13.7 (4.1) ^†^	0.004
MMSE	2016	25.1 (5.3)	21.0 (5.2) ^‡^ ^♯^	27.2 (2.4)^†^ ^♯^	28.9 (1.7) ^†^ ^‡^	<0.001
CDR (Sum of Boxes)	2010	2.9 (3.8)	6.1 (3.5) ^‡^ ^♯^	1.1 (0.8)^†^ ^♯^	0.0 (0.1) ^†^ ^‡^	<0.001
GDS (30 item)	1718	5.0 (4.8)	5.6 (4.8) ^♯^	6.7 (5.8)^♯^	3.9 (4.1) ^†^ ^‡^	<0.001
COWA	1939	8.6 (3.6)	7.3 (3.4) ^‡^ ^♯^	8.3 (3.1)^†^ ^♯^	10.1 (3.3) ^†^ ^‡^	<0.001
Boston Naming Test	1976	8.2 (4.2)	6.5 (3.6) ^‡^ ^♯^	8.2 (3.8)^†^ ^♯^	9.9 (4.3) ^†^ ^‡^	<0.001
WMS LM II	1863	8.2 (4.8)	3.7 (2.2) ^‡^ ^♯^	8.4 (3.4)^†^ ^♯^	12.2 (3.1) ^†^ ^‡^	<0.001
WMS VR II	1649	9.2 (4.5)	4.6 (2.7) ^‡^ ^♯^	8.9 (3.1)^†^ ^♯^	12.0 (3.3) ^†^ ^‡^	<0.001
DST	1433	9.0 (3.1)	8.2 (3.1) ^♯^	8.7 (2.6)^♯^	10.1 (3.1) ^†^ ^‡^	<0.001
IADL (Summed)	1500	10.4 (5.0)	15.0 (6.0) ^‡^ ^♯^	8.4 (2.3)^†^	7.9 (1.0) ^†^	<0.001
Complete Cases	1244					

CDR = Clinical Dementia Rating scale; COWA = Controlled Oral Word Association Test; DST = Digit Span Test; GDS = Geriatric Depression Scale; IADL = Instrumental Activities of Daily Living; MMSE = Mini-mental State Exam; SD = standard deviation; WMS LM II = Weschler Memory Scale: Delayed Logical Memory; WMS VR II = Weschler Memory Scale: Delayed Visual Reproduction.

^†^ p < 0.05 vs. AD by Tukey’s HSD for unequal n’s.

^‡^ p < 0.05 vs. MCI by Tukey’s HSD for unequal n’s.

^♯^ p < 0.05 vs. Controls by Tukey’s HSD for unequal n’s.

### 3.1 Confirmatory factor analysis (CFA)

**Fig 1:**
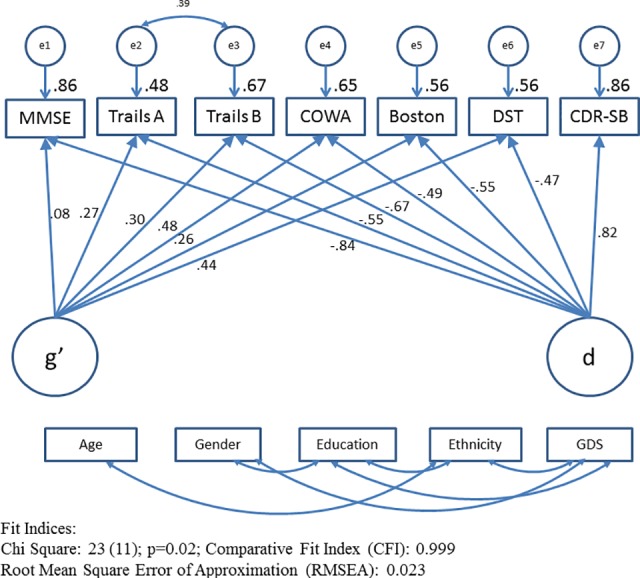
Confirmatory Factor Analysis. All observed variables were adjusted for age, gender, education, ethnicity and geriatric depression scale (GDS) scores (paths not shown for clarity). Abbreviations: BOSTON, Boston Naming Test; CDR-SB, Clinical Dementia Rating scale sum of boxes; COWA, Controlled Oral Word Association Test; DST, Digit Span Test; MMSE, Mini-Mental Status Examination; Trails A, Trail Making Test A; Trails B, Trail Making Test B.

CFA is the foundation of SEM because all latent variable analyses rely on a sound measurement model. In CFA, we built two factor model where factor structure is restricted a priori according to our prior studies. Fig. 1 displays factor structure with factor loadings. Factor loadings are the regression slopes for predicting the indicators from the factor. In this model, functional status is used as an indicator of a latent variable, rather than a correlate. This effectively dissects the shared variance across the cognitive measures (i.e. g) into a larger fraction unrelated to functional status (g’) and a smaller fraction (d).

Data from our study population is then compared with this theoretical model. Fit indices indicate the degree of correspondence or “goodness of fit” between proposed model and empirical data. Our two factor model provides good fit to the data (χ2 = 23.00(11), p = 0.018; RMSEA = 0.023, CFI = 0.999). The model χ2 (p<0.05) rejects the null hypothesis that the data and model implied variance-covariance matrices do not differ. However, χ2 is highly sensitive to sample size and routinely rejects the models involving large samples [47], we relied more on fit indices (RMSEA, CFI) which are insensitive to sample size. CFI (measure the increase in fit relative to baseline model) close to 1.0 and RMSEA (measure the extent to which the specified model of interest reproduces the sample covariance matrix) below 0.05 indicate excellent fit. Thus, this CFA model was used as baseline model when testing the effect of IL-15 levels in the MIMIC model.

In Fig. 1, the numbers on arrows from the latent variable to observed variables are standardized factor loadings (regression weights). These regression coefficients can be interpreted as indicators of validity of the latent variables. Both variables have significant structural coefficients indicating good construct validity. d is significantly related to functional status (CDR- SB) (r = .82, p<0.001) and negatively correlated to cognitive tests performance with statistically significant factor loadings ranging from -.47 to -.67. Higher d reflects greater cognitive impairments. g’ exhibits positive correlation with cognitive test performance with significant factor loading between .26 and .48.

### 3.2 MIMIC model

In the second step, we added the structural part of the model to the measurement model and estimated the MIMIC model. Similar to CFA model, this model fit the data well even though the chi-square test (χ2 = 85.196(25); p ≤ 0.001) rejects it because of large sample size (N = 2016). The alternative fit indices (CFI = 0.995; RMSEA = 0.035) confirm good data-model fit.

Age, gender, education, ethnicity, depression and batch effect of IL-15 measurement were adjusted for their potential confounding effects on observed variables. These covariates act as control variables in our analysis.

**Fig 2:**
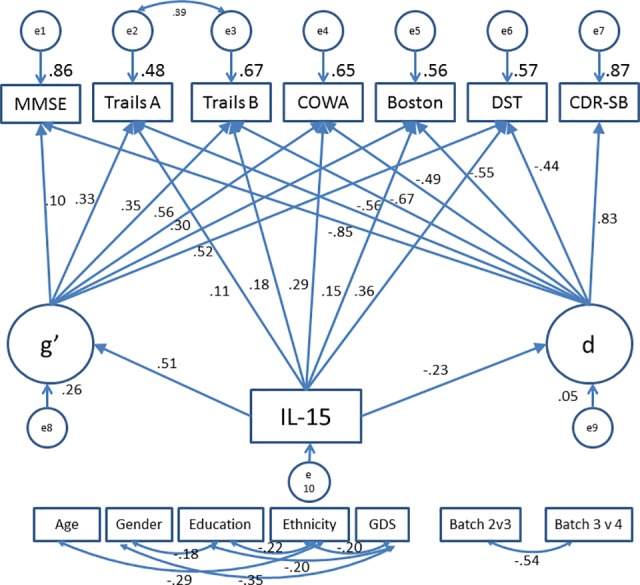
MIMIC Model. All observed variables were adjusted for age, gender, education, ethnicity and geriatric depression scale (GDS) scores (paths not shown for clarity). Abbreviations: BOSTON, Boston Naming Test; CDR-SB, Clinical Dementia Rating scale sum of boxes; COWA, Controlled Oral Word Association Test; DST, Digit Span Test; IL-15, Interleukin-15; MMSE, Mini-Mental Status Examination; Trails A, Trail Making Test A; Trails B, Trail Making Test B. Fit Indices: Chi Square: 85.196 (25); p ≤ 0.001; Comparative Fit Index (CFI): 0.995; Root Mean Square Error of Approximation (RMSEA): 0.035.

All standardized loadings show that they are satisfactory indicators for the latent constructs of cognitive impairments (Fig. 2) (Table 2). Coefficients for the structural paths are interpreted in the same way as regression coefficients. The standardized coefficient value of -.51 for the path from IL-15 to g’ suggests that as serum level of IL-15 rises by one standard deviation, g’ is expected to decrease by .51 standard deviation holding d constant. Similarly, the standardized coefficient value of -.23 for the path from IL-15 to d suggests that with rise of serum level of IL-15 by one standard deviation, d is expected to decrease by .23 standard deviation with g’ held constant.

**Table 2:** Loading/effect of IL-15 on latent variables.

Latent variables	Unstandardized estimate	Standardized Estimate	Standard error	p value
g’	-.905	-.508	.340	.008
d	-2.375	-.234	.371	<.005

Influence of serum IL-15 on cognitive task performances (direct as well as indirect, mediated by g’ and d) was estimated simultaneously via standardized partial regression coefficients. None of the direct path from IL-15 to individual cognitive test was found significant. Thus, the cumulative effect of IL-15 on cognitive test performance is mediated by g’ and d which represent significant independent determinants of observed cognitive performance.

The cumulative effect of IL-15 on the cognitive variance is contrasting. Negative and significant correlation of IL-15 with d, translates to positive correlation with cognitive variance extracted by d whereas, IL-15’s effect on the cognitive variance represented by g’ is negative. In other words, elevated IL-15 levels correlate with lesser dementia related cognitive impairment and higher cognitive impairments which are not dementia related. Thus, IL-15’s effect on cognitive performance through g’ is adverse, while its effect on cognition through d is protective. These effects sum as determinants of observed cognitive performance, and potentially counteract each other. However, d is uniquely related to dementia severity (i.e., CDR-SB) by definition, and empirically to clinicians’ diagnoses (by ROC). Thus, IL-15’s protective effects on cognitive performance through d are arguably more clinically salient, and its total summed effects on cognitive performance appear to be split across clinically salient and irrelevant compartments of observed cognitive measure variance.

## Discussion

In literature, IL-15 has been shown to be associated with both cognitive impairments and neuropsychiatric symptoms in AD but those results were not convincing [16,17,25,26]. Our analysis which has several strengths relative to earlier studies reveals some of the complexity in their association. It is the largest study to date of IL-15’s association with cognitive performance in humans and we have associated IL-15 with a latent dementia phenotype rather than with categorical diagnoses. This minimizes the potential measurement error associated with cognitive performance measures and retains information lost in categorical classifications [48,49]. Other factors potentially affecting our results is use of serum biomarkers, as opposed to CSF or other tissues.

We have found that IL-15 has significant effects on cognition, but they are exclusively mediated by latent constructs g’ and d. Independent of those paths, IL-15 has no direct effects on cognitive performance. g’ and d in turn are non-overlapping (“orthogonal) fractions of Spearman’s general intelligence factor “g”. g contributes variance to almost every cognitive assessment, and is independent of a measure’s face validity as a measure of domain specific cognitive functions (e.g., “memory”, “executive function”, etc.). Thus, IL-15’s association with multiple cognitive measures in this model appears to be unrelated to the individual cognitive domains they purport to measure. We have noted a similar constraint on the association between a second serum protein, VDBP and cognition [30], and suspect that it may be a general feature of the interactions between serum biomarkers and cognitive performance.

d (but not g’) has been previously associated with atrophy in the Default Mode Network (DMN) [29]. The DMN in turn has been implicated in AD through a-beta deposition [50] and its invasion by paired helical filament tauopathy, a precursor to neurofibrillary tangle (NFT) formation [50]. This suggests that serum cytokines may be effecting changes in network structure or function. Such changes may mediate their observed associations with cognitive performance, and hence dementia. Alteration of blood brain barrier alone may not be responsible for the transport of cytokines to/from the site of neuroinflammation. Instead, homeostatic mechanisms present at the brain barriers, such as their secretome and receptor-mediated signaling [51] or local expression of cytokines by activated microglia may also play the role. Microglial IL-15 was found to play a pivotal role in the neuroinflammation in mouse [21].

Dementia syndromes including AD are known to have circumscribed pattern of neurodegeneration which reflects the intrinsic functional network structure [52,53]. This ‘‘network degeneration hypothesis’’ has been validated by animal models, neuropathological and neuroimaging findings [54]. Seeley et al mapped the functional and structural networks in five different dementia syndromes including AD and each network was robust, convergent and anatomically predictable [55]. AD affects connectivity in several resting state networks (e.g. “salience” network), not only the DMN [56–59]. Thus, if IL-15 affects DMN structure or function through d, its effect on g’ may be representative of changes in entirely distinct but unknown networks.

IL-15’s effects on observed cognitive performance are independently mediated by g’ and d and appear to be contradictory. However, they represent the independent contributors to the observed cognitive performance. This helps explain the variability in findings across other investigators. Hall and Rentzos found negative association with IL-15 and AD using categorical diagnoses where g’, being a dominant contributor to cognitive performance (thus, the clinical diagnosis) may have masked the associations mediated by d. d’s associations with observed cognitive performance are relatively strong, but IL-15’s association with d itself is relatively weak (compared to its association with g’). Thus, both paths account for roughly equal proportions of variance in observed cognitive performance, and might potentially “cancel each other out.” Regardless, they may be affecting independent compartments of variance, each mediated by distinct neural networks. Only d’s effects are functionally salient. IL-15’s effects on cognitive performance via d should protect against dementia, even as its effects via g’ impair cognitive performance. This is because d’s effect on cognition is manifestly more salient to dementia status than is g’s.

How might a serum cytokine effect discrete and divergent change in a network’s structure or function? IL-15 is known to be proinflammatory cytokine. In the event of CNS injury, it plays a role in early inflammatory responses and glial cell activation [60,61]. Eventual result is the reactive gliosis after CNS injury which is evident by reduction of the reactivity of both astrocytes and microglia by a blocking antibody against IL-15 in the brain after lipopolysaccharide (LPS) treatment [62]. In cultured microglia, IL-15 blockade reduces the activation of proinflammatory mediators like mitogen-activated protein kinases (MAPK) and nuclear factor (NF)-κB ([60]. Microglial activation and microglial derived oxidative stress leading to neurodegeneration have been described as critical processes in AD pathogenesis [63].

However, IL-15 can be a neuroprotective agent by virtue of its anti-apoptotic and neurotrophic properties. IL-15 in the brain functions as potent chemo-attractant and leads to T cells trafficking at the site of injury and T cell accumulation has been associated with neuroprotection (IL-15 promotes the survival of natural killer cells and γδ-T lymphocytes) at certain brain areas [64]. Huang et al found impaired T cells migration to injured facial motor nucleus in mice deficient in IL-15 and IL-15Rα leading to neurodegeneration [65]. IL-15 and IL-2 induce distinctive levels of signaling through common receptor subunits but interestingly, the biological responses mediated by IL-2 and IL-15 are highly distinct as illustrated by the fate of antigen-activated T cells.

There is evidence in literature that differences in susceptibility to inflammatory effects could be related to differences in the microglial cell density within specific brain regions [66]. In AD, Aβ plaques are frequently associated with both reactive astrocytes and activated microglia, and there is striking overlap between the spatial distribution of amyloid-β pathology and the default mode network [67]. Moreover, the blood brain barrier (BBB) has been described to be more permeable for cellular infiltrations at sites of high microglial density [68] which further allow accumulation of monocytes eventually differentiating into microglia [69,70]. Thus, we could speculate that in the event of higher density of microglia and a more permeable BBB at sites corresponding to DMN, Aβ could invoke an inflammatory response and affect production of inflammatory mediators like IL-15.

The intrathecal production of IL-15 in the basal state is contributed to by both neurons and glia but in the event of inflammatory and autoimmune challenge, the source of IL-15 includes both infiltrating and residential cells [71]. Additionally, circulating IL-15 from the peripheral blood may contribute to the neuro-inflammatory response. The central nervous system (CNS) and the immune system form a communication network via which immune mediators inform the brain about events in the body and brain effects function of immune cells. On that line, IL-15 can cross the BBB to reach the brain when there is an inflammation. The BBB responds to inflammation with increased cytokine production, activation of transport proteins, increased permeability, and selective upregulation of cytokine transport [72,73]. These pathological alterations of BBB can be topographically heterogeneous. Experimental neuro-inflammation (from LPS) resulted in enhanced CNS uptake of blood-borne IL-15, and this was associated with further increase in BBB permeability to the vascular markers. In most brain cells the actions of IL-15 are mediated through the JAK/STAT pathway, whereas in brain microvessel endothelial cells IL-15 can induce both STAT and nuclear factor (NF)-κB [74]. Endothelial signaling is an integral part of the BBB response to cytokines, and it plays an essential role in modulation of CNS functions. The regulatory changes of the BBB are an integral part of CNS inflammation and autoimmune disease, thereby providing a potential target for therapeutic intervention. In a mice study, Pan et al concluded that that IL-15 is a novel mediator of TNF signaling at the level of the BBB, serving to amplify and modulate TNF signaling [75]. TNF treatment increased the level of expression of IL-15 receptors in cerebral microvessels. Together with TNF, peripheral sources of IL-15 can induce NFκB signaling and alter BBB structure and functions. Since upregulation of the IL-15 system is a major inflammatory response of the BBB endothelia to TNF, IL-15 signaling appears to provide feedback control for TNF function.

IL-15 has the potential to contribute to neurogenesis during neuropathological states, although it is not certain how this affects the AD pathology. It has impact on neural stem cells (NSC) proliferation and differentiation predominantly exists in subventricular zone (SVZ) and dentate gyrus (DG) of adult human brain [76]. By affecting neural cell differentiation via a signal transduction pathway involving IL-15Rα and STAT3 as the signal transduction alters microtubule-associated protein 2 (MAP-2) protein levels IL-15 affects neuronal differentiation from NSCs [77]. In vitro, IL-15 deficiency results in a defective activation of both the janus kinase/signal transducers and activators of transcription (JAK/STAT) and the ERK/MAPK pathways in adult NSCs, which are key regulators of NSC proliferation and differentiation [78]. Consequently, the effect of IL-15 upon these pathways may be accountable for the maintenance of self-renewal as well as the proliferative capabilities of NSCs within the adult brain. Human post-mortem analysis, animal models and *in vitro* studies incline towards progressive impairment of neuroregeneration resources in AD [79]. However, some evidence for stimulating growth factors restores some of the cognitive loss and ameliorates behavioral skills, suggests that AD brain does have neuroregenerative potential. IL-15, doing so may contribute to preserving the d related cognitive impairments.

In summary, we have been able to associate serum levels of IL-15 with dementing process of AD as well as non-dementing cognitive impairment. IL-15’s association with cognition is complex, particularly the conflicting correlation mediated by latent constructs. The intermediary latent constructs may act as proxies for network structures and/or functions. Thus, a latent variable approach may be necessary to the comprehensive assessment of biomarker /cognition associations. These findings warrant attention and further research to determine the corresponding changes in structural and functional networks.
